# Commentary: Experimental evidence for compositional syntax in bird calls

**DOI:** 10.3389/fpsyg.2016.01171

**Published:** 2016-08-03

**Authors:** Steven Phillips, William H. Wilson

**Affiliations:** ^1^Mathematical Neuroinformatics Group, Human Informatics Research Institute, National Institute of Advanced Industrial Science and TechnologyTsukuba, Japan; ^2^School of Computer Science and Engineering, University of New South WalesSydney, NSW, Australia

**Keywords:** systematicity, compositionality, category theory, syntax, universality

Suzuki et al. (2016) report a remarkable discovery: the first evidence of a combinatorial syntax and semantics in non-humans; specifically, Japanese great tits. However, remarkable discoveries require remarkable evidence. Their data provide impressive support for a compositional syntax. Yet, evidence for compositionality is not necessarily evidence for one of the hallmarks of human language and thought: *systematicity*—a structural equivalence relation over cognitive capacities (Fodor and Pylyshyn, 1988). Some versions of compositionality support systematicity and some do not (Aizawa, 2003; Phillips and Wilson, 2010). We surmise that the question remains open as to whether the version of compositionality that is evident in the bird calls study does indeed support systematicity. Drawing on a theory of systematicity (Phillips and Wilson, 2010) we derive testable criteria for systematicity in the context of bird calls. These criteria must be met before claims of *human-like* compositional syntax in non-humans could be justified.

Systematicity is a property of (some core aspects of) human language and thought whereby having the capacity to understand certain expressions or situations implies having the capacity to understand certain other, structurally related, expressions/situations (Fodor and Pylyshyn, 1988). The archetypal example of systematicity is where one has the capacity to understand the sentence “John loves Mary” if and only if one has the capacity to understand the sentence “Mary loves John,” assuming one also understands the constituents John, loves, and Mary, where the common structural relation between entities John and Mary is love. Other forms of systematicity follow from the systematic nature of thought, generally. For example, in reasoning, if one is told that John and Mary went to the store, then one can infer that John went to the store—*P* and *Q* implies *P* (see Fodor and Pylyshyn, 1988; McLaughlin, 2009). Hence, systematicity is a central property of human language and thought that warrants investigation in non-humans if the evolutionary story is to be properly told.

The authors' claim of a compositional syntax and semantics for Japanese great tits aligns with some aspects of the classical (symbol systems) notion of compositionality, which is sometimes called *classical compositionality*. The experiments revealed that great tits extracted different meanings for notes and their syntactic compositions: an ABC note means “scan for danger,” a D note means “approach the caller,” and their syntactic combination ABC-D means “scan for danger then approach the caller,” whereas the (agrammatical) combination D-ABC has no meaning. The classical compositionality account says that the meaning of a complex utterance is understood from the meanings of the constituent utterances and the correspondence between syntactic relationships among those constituents and their semantic relationships. In the case of great tits, the meaning of bird call ABC-D (scan, then advance) is understood from the meanings of the constituent calls ABC (scan) and D (advance) and the correspondence between their syntactic relationship (ABC is-followed-by D) and their corresponding semantic relationship (scanning is-followed-by advancing). Notice, importantly, that the common structural relation in the bird calls case is not simply temporal order, as evidenced by the counterexample, D-ABC. Accordingly, classical compositionality includes typing (relational role) information that determines the allowable syntactic constructions, which are supposed to be aligned with the corresponding semantic structural relations (see below for further examples). Failure to make this distinction may be seen as one reason why simple associative/statistical models that are based on co-occurrence relations among the constituents of complex capacities (e.g., words in sentences) fail to account for the complexity and subtlety of human language and thought (Fodor and Pylyshyn, 1988; Everaert et al., 2015).

The point of departure from the authors' claim of compositionality and the notion of classical compositionality in humans is in regard to systematicity. A demonstration of systematicity requires evidence that the capacity for two structurally equivalent abilities is *indivisible*. For the *loves* example, such a demonstration involves evidence that the capacity to understand “John loves Mary” is equivalent to the capacity to understand “Mary loves John,” because these two capacities share the same syntactic/semantic relation, loves. Put simply, there is no situation of having one capacity, but not the other. Clearly, however, this form of (symmetric) structural similarity is meaningless to a great tit in the context of predator deterrence, presumably because the capacity to understand D-ABC, i.e., advance before scanning for predators, has severe consequences for survival. Similar, nonsensical situations arise in human language. For instance, one can say “John fed hay (to the horse),” but it makes no sense to say “Hay fed John.” Systematicity need not be confined to symmetric structures. Instances of systematicity based on asymmetric structures also exist, for example, where one has the capacity to understand the sentence “John fed hay” if one has the capacity to understand the sentence “Mary fed hay,” assuming that one understands the constituents John, fed, hay and Mary.

An analogous criterion for great tits can also be derived as a test for the systematicity of their “linguistic” ability, using a category theoretic approach to systematicity (Phillips and Wilson, 2010, 2014). Category theory is a branch of mathematics for reasoning about systems of entities and their relationships: a system regarded as a *category* consists of a collection of *objects*, a collection of relations between objects, called *arrows, morphisms*, or *maps*, and a composition operation that composes pairs of (compatible) arrows into other arrows. A category theory approach to modeling a cognitive system is to regard sets of cognitive states as objects, cognitive processes that map states to states as arrows, and composition of cognitive processes as the composition operation. In this way, a category theory explanation for systematicity says that every cognitive capacity in a collection of systematically related capacities is the composition of a *universal* arrow that is common to all capacities in that collection and a unique arrow that is specific to that particular capacity, so one has each and every capacity if and only if one has the universal arrow and a way to compose arrows, which includes the universal arrow with the unique arrows. An advantage of deriving criteria from a category theory perspective on systematicity, as opposed to other approaches, is that it isolates just those properties that are essential (i.e., necessary and sufficient) for systematicity from those properties that are idiosyncratic to the domain at hand. In particular, the characterizations of systematicity introduced above, which were drawn from the original classicists' perspective, presume peripheral (symbolic) capacities that are far beyond those of non-humans, whence it is unclear how systematicity is even testable in non-human cohorts. In contrast, a categorical, “objects and arrows” perspective generalizes the notion of compositionality in a way that affords realistic tests of systematicity in non-humans. A visual/geometric intuition of the formalism that underlies the example, provided next, is given in Figure 1.

**Figure 1:**
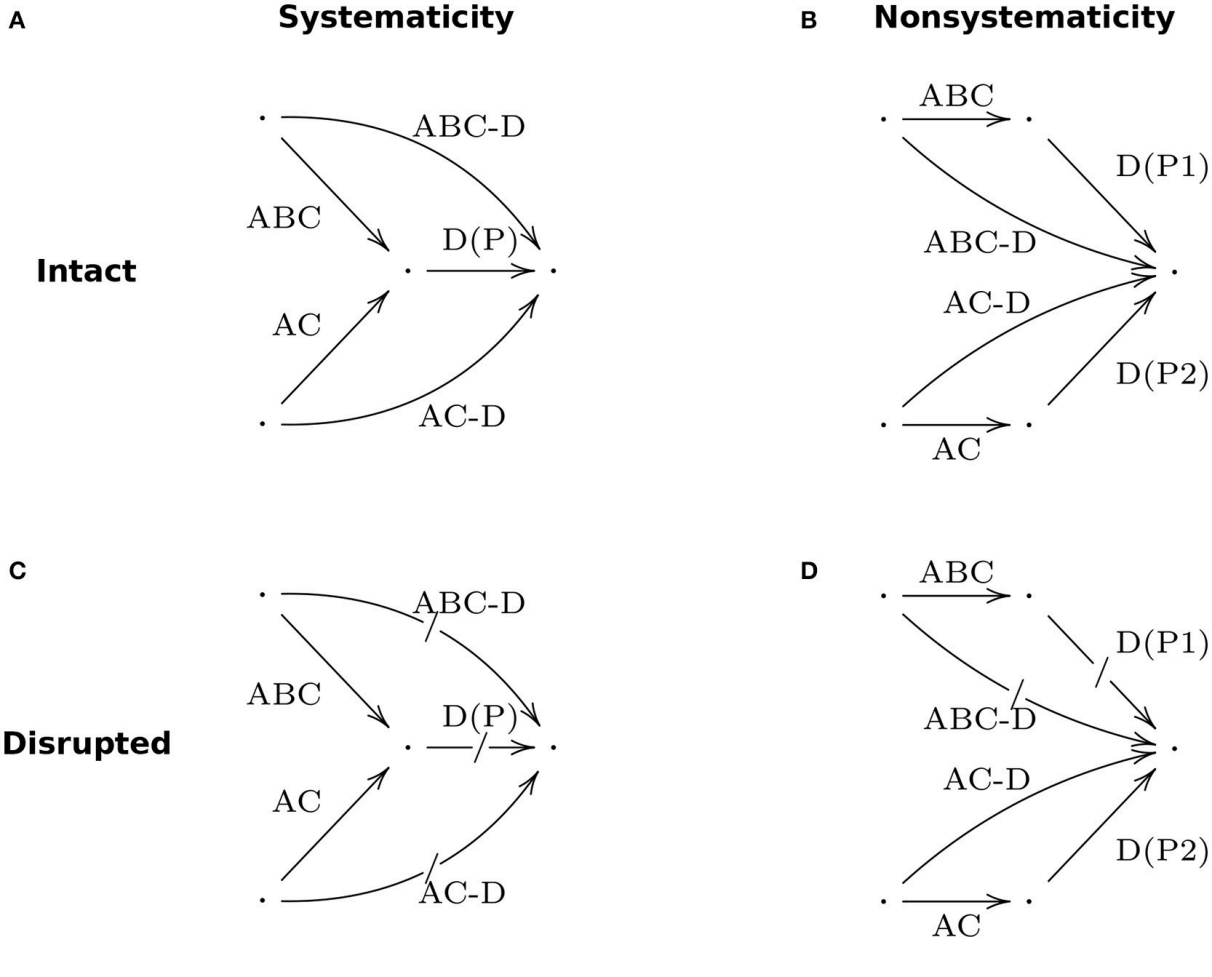
**A category theory perspective on compositionality for ABC-D and AC-D bird calls for systematic (A) and nonsystematic (B) cases**. Arrow labels correspond to constituent and compositional capacities. Disruption of the constituent arrow for capacity D for the systematic composition **(C)** results in disruption of the capacities ABC-D and AC-D calls (indicated by slashed arrows). Disruption of the constituent arrow for capacity D(P1) for the nonsystematic composition **(D)** results in the disruption of capacity ABC-D, but not capacity AC-D.

The authors explain that great tits have a variety of calls associated with different predators, such as AC-D and BC-D. Then an instance of systematicity is when a great tit demonstrates the capacity to understand the ABC-D calls if and only if it demonstrates the capacity to understand the structurally-related AC-D or BC-D calls. Because systematicity is a structural equivalence relation over capacities (McLaughlin, 2009), demonstrating that a bird understands both ABC-D and AC-D calls is only half of the criteria for systematicity in this example. One must also demonstrate that there exists a component process that when absent or disrupted results in the absence or disruption of both ABC-D and AC-D capacities, not the exclusive disruption of just one or the other capacity. Naturally, this criterion for systematicity extends to situations of having more than two structurally related capacities, e.g., ABC-D, AC-D, and BC-D. The case where only one capacity is disrupted, say ABC-D but not AC-D, is evidence against the kind of compositionality possessed by humans. The essential problem to be addressed empirically is that there are two ways to realize capacities ABC-D and AC-D: (1) via a shared component process P that realizes constituent capacity D, and (2) via distinct component processes P1 and P2 that separately realize constituent capacity D for the capacities ABC-D and AC-D, respectively. In the first case, disruption of P implies disruption of both capacities—systematicity. In the second case, disruption of P1 implies disruption of the ABC-D capacity, but not the AC-D capacity, since the component process P2 is intact—no systematicity.

As the authors point out, studies of language-like behavior in non-humans are important to establish the missing link in the evolutionary story of human language. Systematicity is afforded by effective (re)use of cognitive resources, as the categorical perspective highlights. The potential relationship between cognitive resource use (cost) and cognitive capacity to successfully interact with the environment (benefit) leads naturally to important questions regarding the extent that compositionality is driven by environmental forces vs. genetic good fortune (Hauser et al., 2002), or some combination of these. For instance, one can envisage situations where the lack of variability in the environment places little demand for a systematic compositional syntax—e.g., small variation in predator types, whereby each situation is represented without representing the common structural relations (nonsystematic compositionality). Alternatively, environments filled with different types of predators requiring different types of related responses may drive systematic compositionality. Such situations would suggest that systematic compositionality is driven by environmental forces. However, systematicity in the absence of diverse environmental contingencies suggests fortuitous genetic endowment, in which case the environment plays a lesser role. Empirical data that dissociate systematic from nonsystematic compositionality in other species is evidence of a branch point in the evolution of human language and thought, a hallmark of which is the sheer diversity of situations that are within the capacity of our cognitive system.

## Author contributions

All authors listed, have made substantial, direct and intellectual contribution to the work, and approved it for publication.

## Funding

This work was supported by a Japanese Society for the Promotion of Science Grant-in-aid (26280051).

### Conflict of interest statement

The authors declare that the research was conducted in the absence of any commercial or financial relationships that could be construed as a potential conflict of interest.
